# Semidefinite bounds for nonbinary codes based on quadruples

**DOI:** 10.1007/s10623-016-0216-5

**Published:** 2016-05-11

**Authors:** Bart Litjens, Sven Polak, Alexander Schrijver

**Affiliations:** Korteweg-De Vries Institute for Mathematics, University of Amsterdam, Amsterdam, The Netherlands

**Keywords:** Code, Nonbinary code, Upper bounds, Semidefinite programming, Delsarte, 94B65, 05E10, 90C22, 20C30

## Abstract

For nonnegative integers *q*, *n*, *d*, let $A_q(n,d)$ denote the maximum cardinality of a code of length *n* over an alphabet [*q*] with *q* letters and with minimum distance at least *d*. We consider the following upper bound on $A_q(n,d)$. For any *k*, let $\mathcal{C}_k$ be the collection of codes of cardinality at most *k*. Then $A_q(n,d)$ is at most the maximum value of $\sum _{v\in [q]^n}x(\{v\})$, where *x* is a function $\mathcal{C}_4\rightarrow {\mathbb {R}}_+$ such that $x(\emptyset )=1$ and $x(C)=\!0$ if *C* has minimum distance less than *d*, and such that the $\mathcal{C}_2\times \mathcal{C}_2$ matrix $(x(C\cup C'))_{C,C'\in \mathcal{C}_2}$ is positive semidefinite. By the symmetry of the problem, we can apply representation theory to reduce the problem to a semidefinite programming problem with order bounded by a polynomial in *n*. It yields the new upper bounds $A_4(6,3)\le 176$, $A_4(7,3)\le 596$, $A_4(7,4)\le 155$, $A_5(7,4)\le 489$, and $A_5(7,5)\le 87$.

## Introduction

Let ${\mathbb {Z}}_+$ denote the set of nonnegative integers, and denote $[m]=\{1,\ldots ,m\}$, for any $m\in {\mathbb {Z}}_+$. Fixing $n,q\in {\mathbb {Z}}_+$, a *code* is a subset of $[q]^n$. So [*q*] serves as the alphabet and *n* as the word length. We will assume throughout that $q\ge 2$. (If you prefer $\{0,1,\ldots ,q-1\}$ as alphabet, take the letters mod *q*.) While this paper is mainly meant to handle the case $q\ge 3$, the results also hold for $q=2$.

For $v,w\in [q]^n$, the *(Hamming) distance* $d_H(v,w)$ is equal to the number of $i\in [n]$ with $v_i\ne w_i$. The *minimum distance* of a code *C* is the minimum of $d_H(v,w)$ taken over distinct $v,w\in C$. Then $A_q(n,d)$ denotes the maximum cardinality of a code with minimum distance at least *d*. We will study the following upper bound on $A_q(n,d)$, sharpening Delsarte’s classical linear programming bound [4].

For $k\in {\mathbb {Z}}_+$, let $\mathcal{C}_k$ be the collection of subsets *C* of $[q]^n$ with $|C|\le k$. For each $x:\mathcal{C}_4\rightarrow {\mathbb {R}}$ define the $\mathcal{C}_2\times \mathcal{C}_2$ matrix *M*(*x*) by

1 $$\begin{aligned} M(x)_{C,C'}:=x(C\cup C') \end{aligned}$$

for $C,C'\in \mathcal{C}_2$. Then define

2 $$\begin{aligned} B_q(n,d):=\displaystyle \max _x\sum _{w\in [q]^n}x(\{w\}), \hbox { where } x:\mathcal{C}_4\rightarrow {\mathbb {R}}_+ \hbox { satisfies} \end{aligned}$$

(i) $$x(\emptyset )=1$$,
(ii) $x(C)=0$ if the minimum distance of *C* is less than *d*,
(iii) *M*(*x*) is positive semidefinite.

### **Proposition 1**

$$A_q(n,d)\le B_q(n,d)$$.

### *Proof*

Let $D\subseteq [q]^n$ have minimum distance at least *d* and satisfy $|D|=A_q(n,d)$. Define $x:\mathcal{C}_4\rightarrow {\mathbb {R}}$ by $x(C)=1$ if $C\subseteq D$ and $x(C)=0$ otherwise. Then *x* satisfies the conditions: (iii) follows from the fact that for this *x* one has $M(x)_{C,C'}=x(C)x(C')$ for all $C,C'\in \mathcal{C}_2$. Moreover, $\sum _{w\in [q]^n}x(\{w\})=|D|=A_q(n,d)$. $\square$

The optimization problem (2) is huge, but, with methods from representation theory, can be reduced to a size bounded by a polynomial in *n*, with entries (i.e., coefficients) being polynomials in *q*. This makes it possible to solve (2) by semidefinite programming for some moderate values of *n*, *d*, and *q*, leading to improvements of best known upper bounds for $A_q(n,d)$.

To explain the reduction, let *H* be the wreath product $S_q^n\rtimes S_n$. For each *k*, the group *H* acts naturally on $\mathcal{C}_k$, maintaining minimum distances and cardinalities of elements of $\mathcal{C}_k$ (being codes). Then we can assume that *x* is invariant under the *H*-action on $\mathcal{C}_4$. That is, we can assume that $x(C)=x(D)$ whenever $C,D\in \mathcal{C}_2$ and $D=g\cdot C$ for some $g\in H$. Indeed, (2)(i)(ii)(iii) are maintained under replacing *x* by $g\cdot x$. (Note that $M(g\cdot x)$ is obtained from *M*(*x*) by simultaneously permuting rows and columns.) Moreover, the objective function does not change by this action. Hence the optimum *x* can be replaced by the average of all $g\cdot x$ (over all $g\in H$), by the convexity of the set of positive semidefinite matrices. This makes the optimum solution *H*-invariant.

Let $\Omega$ be the set of *H*-orbits on $\mathcal{C}_4$. Note that $\Omega$ is bounded by a polynomial in *n* (independently of *q*). As there exists an *H*-invariant optimum solution, we can replace, for each $\omega \in \Omega$ and $C\in \omega$, each variable *x*(*C*) by a variable $y(\omega )$. In this way we obtain *M*(*y*).

Then *M*(*y*) is invariant under the action of *H* on its rows and columns, induced from the action of *H* on $\mathcal{C}_2$. Hence *M*(*y*) can be block-diagonalized by $M(y)\mapsto U^\mathsf{T}M(y) U$, where *U* is a matrix independent of *y*. The entries in each block are linear functions of the variables $y(\omega )$. There are several equal (or equivalent) blocks. Taking one block from each such class gives a matrix of order polynomial in *n* with numbers that are polynomials in *q*. The issue crucial for us is that the original matrix *M*(*y*) is positive semidefinite if and only if each of the blocks is positive semidefinite.

In this paper we will describe the blocks that reduce the problem. With this, we found the following improvements on the known bounds for $A_q(n,d)$, with thanks to Hans D. Mittelmann for his help in solving the larger-sized problems.

**Table:** 

*q*	*n*	*d*	Best lower bound known	New upper bound	Best upper bound previously known
4	6	3	164	176	179
4	7	3	512	596	614
4	7	4	128	155	169
5	7	4	250	489	545
5	7	5	53	87	108

The best upper bounds previously known for $A_4(6,3)$ and $A_4(7,3)$ are Delsarte’s linear programming bound [4]; the other three best upper bounds previously known were given by Gijswijt, Schrijver, and Tanaka [7]. We refer to the most invaluable tables maintained by Andries Brouwer [3] with the best known lower and upper bounds for the size of error-correcting codes (see also Bogdanova, Brouwer, Kapralov, and Östergård [1] and Bogdanova and Östergård [2] for studies of bounds for codes over alphabets of size $q=4$ and $q=5$, respectively).

### Comparison with earlier bounds

The bound $B_q(n,d)$ described above is a sharpening of Delsarte’s classical linear programming bound [4]. The value of the Delsarte bound is equal to our bound after replacing $\mathcal{C}_4$ and $\mathcal{C}_2$ by $\mathcal{C}_2$ and $\mathcal{C}_1$, respectively, which generally yields a less strict bound.

We can add to (2) the condition that, for each $D\in \mathcal{C}_4$, the $S(D)\times S(D)$ matrix

3 $$\begin{aligned} (x(C\cup C'))_{C,C'\in S(D)} \hbox { is positive semidefinite}, \end{aligned}$$

where $S(D):=\{C\in \mathcal{C}_4\mid C\supseteq D, |D|+2|C\setminus D|\le 4\}$. (So (iii) in (2) is the case $D=\emptyset$.) Also the addition of (3) allows a reduction of the optimization problem to polynomial size as above. (It can be seen that adding (3) for $|D|=2$ suffices.) For $q=2$ we obtain in this way the bound given by Gijswijt et al. [6]. Our present description gives a more conceptual and representation-theoretic approach to the method of [6].

A bound intermediate to the Delsarte bound and the currently investigated bound is based on considering functions $x:\mathcal{C}_3\rightarrow {\mathbb {R}}_+$ and the related matrices—see Schrijver [9] for binary codes and Gijswijt et al. [7] for nonbinary codes.

## Preliminaries on representation theory

We assume some familiarity with classical representation theory, in particular of the symmetric group $S_n$ and of finite groups in general. In this section we give a brief review, also to settle some notation and terminology. We refer to Sagan [8] for background.

A group *G* *acts* on a set *X* if there is a group homomorphism $G\rightarrow S_X$, where $S_X$ is the group of bijections $X\rightarrow X$. The image of $g\in G$ in $S_X$ is indicated by $g\cdot$. If *X* is a linear space, the bijections are assumed to be linear functions. The action of *G* on a set *X* induces an action of *G* on the linear space ${\mathbb {C}}^X$, by $(g\cdot f)(x):=f(g^{-1}\cdot x)$ for all $g\in G$, $f\in {\mathbb {C}}^X$, and $x\in X$. If any group *G* acts on *X*, then $X^G$ denotes the set of elements of *X* invariant under the action of *G*.

Let $m\in {\mathbb {Z}}_+$ and let *G* be a finite group acting unitarily on $V={\mathbb {C}}^m$ (meaning that for each $g\in G$ there is a unitary $m\times m$ matrix *U* such that $g\cdot x=Ux$ for all $x\in {\mathbb {C}}^m$). Then *V* can be decomposed uniquely as direct sum of the *G*-isotypical components $V_1,\ldots ,V_k$. For distinct *i*, *j*, $V_i$ and $V_j$ are orthogonal (with respect to the inner product $\langle x,y\rangle =x^*y$ for $x,y\in {\mathbb {C}}^m$, where $x^*$ is the conjugate transpose of *x*). Next, each $V_i$ is a direct sum $V_{i,1}\oplus \cdots \oplus V_{i,m_i}$ of mutually *G*-isomorphic, irreducible *G*-modules, in such a way that $V_{i,j}$ and $V_{i,j'}$ are orthogonal for distinct $j,j'$. (This decomposition is generally not unique.) For each $i\le k$ and $j\le m_i$, choose a nonzero $u_{i,j}\in V_{i,j}$ such that for each *i* and all $j,j'\le m_i$ there exists a *G*-isomorphism $V_{i,j}\rightarrow V_{i,j'}$ bringing $u_{i,j}$ to $u_{i,j'}$. For each $i\le k$, let $U_i$ be the matrix $[u_{i,1},\ldots ,u_{i,m_i}]$, considering the $u_{i,j}$ as columns. We call any matrix set $\{U_1,\ldots ,U_k\}$ that can be obtained in this way *representative* for the action of *G* on ${\mathbb {C}}^m$. It has the property that the function

4 $$\begin{aligned} \Phi :({\mathbb {C}}^{m\times m})^G\rightarrow \bigoplus _{i=1}^k{\mathbb {C}}^{m_{i}\times m_i} \quad \text { with } \quad \Phi (X):=\bigoplus _{i=1}^{k}U_{i}^* XU_i \end{aligned}$$

for $X\in ({\mathbb {C}}^{m\times m})^G$ is bijective. So $\sum _im_i^2$ is equal to the dimension of $({\mathbb {C}}^{m\times m})^G$ (and hence can be considerably smaller than *m*).

Another important property of a representative matrix set is that any $X\in ({\mathbb {C}}^{m\times m})^G$ is positive semidefinite if and only if $\Phi (X)$ is positive semidefinite. (A positive semidefinite matrix is a Hermitian matrix with all eigenvalues nonnegative.)

In our applications below, throughout *G* is acting real-orthogonally on a vector space $V={\mathbb {R}}^m$; that is, for each $g\in G$ there is a real orthogonal $m\times m$ matrix *U* with $g\cdot x=Ux$ for each $x\in {\mathbb {C}}^m$.

Moreover, as it turns out, for the cases considered in the present paper the matrices $U_i$ can be taken real-valued (which is computationally convenient). This implies that $\Phi (X)=\bigoplus _{i=1}^kU_i^\mathsf{T}XU_i$ for $X\in ({\mathbb {R}}^{m\times m})^G$ and $\Phi (({\mathbb {R}}^{m\times m})^G)=\bigoplus _{i=1}^k{\mathbb {R}}^{m_i\otimes m_i}$. Moreover, a matrix $X\in {\mathbb {R}}^{m\times m}$ is positive semidefinite if and only if $U_i^\mathsf{T}XU_i$ is positive semidefinite for each $i=1,\ldots ,k$. For later reference we state that, since for all *i*, *j*, $V_{i,j}$ is the linear space spanned by $G\cdot u_{i,j}$,

5 $$\begin{aligned} {\mathbb {R}}^m=\bigoplus _{i=1}^k\bigoplus _{j=1}^{m_i}{\mathbb {R}}G\cdot u_{i,j}. \end{aligned}$$

It will turn out to be convenient to consider the columns of the matrices $U_i$ as elements of the dual space $({\mathbb {R}}^m)^*$ (by taking the standard inner product). Then each $U_i$ is an ordered set of linear functions on ${\mathbb {R}}^m$. (The order plays a role in describing a representative matrix set for the action of the wreath product $G^n\rtimes S_n$ on $V^{\otimes n}$.)

### A representative set for the action of $S_n$ on $V^{\otimes n}$

Classical representation theory of the symmetric group yields a representative set for the natural action of $S_n$ on $V^{\otimes n}$, where *V* is a finite-dimensional vector space, which we will describe now.

For $n\in {\mathbb {Z}}_+$, $\lambda \vdash n$ means that $\lambda$ is equal to $(\lambda _1,\ldots ,\lambda _t)$ for some *t*, with $\lambda _1\ge \cdots \ge \lambda _t>0$ integer and $\lambda _1+\cdots +\lambda _t=n$. The number *t* is called the *height* of $\lambda$. The *Young shape* $Y(\lambda )$ of $\lambda$ is the set

6 $$\begin{aligned} Y(\lambda ):=\big \{(i,j)\in {\mathbb {Z}}_+^2\mid 1\le j\le t, 1\le i\le \lambda _j\big \}. \end{aligned}$$

For any $j_0\le t$, the set of elements $(i,j_0)$ in $Y(\lambda )$ is called the $j_0$-*th row* of $Y(\lambda )$. Let $R_{\lambda }$ be the group of permutations $\pi$ of $Y(\lambda )$ with $\pi (Z)=Z$ for each row *Z* of $Y(\lambda )$. For any $i_0\le \lambda _1$, the set of elements $(i_0,j)$ in $Y(\lambda )$ is called the $i_0$ *-th column* of $Y(\lambda )$. Let $C_{\lambda }$ be the group of permutations $\pi$ of $Y(\lambda )$ with $\pi (Z)=Z$ for each column *Z* of $Y(\lambda )$.

A $\lambda$-*tableau* is a function $\tau :Y(\lambda )\rightarrow {\mathbb {Z}}_+$. We put $\tau \sim \tau '$ for $\lambda$-tableaux $\tau ,\tau '$ if $\tau '=\tau r$ for some $r\in R_{\lambda }$. A $\lambda$-tableau is *semistandard* if in each row the entries are nondecreasing and in each column the entries are increasing. Let $T_{\lambda ,m}$ denote the collection of semistandard $\lambda$-tableaux with entries in [*m*]. Note that $T_{\lambda ,m}\ne \emptyset$ if and only if $\lambda$ has height at most *m*.

Let $B=(B(1),\ldots ,B(m))$ be an ordered basis of $V^*$. For $\tau \in T_{\lambda ,m}$, define the following element of $(V^*)^{\otimes n}$:

7 $$\begin{aligned} u_{\tau ,B}:=\sum _{\tau '\sim \tau }{\sum _{c\in C_{\lambda }}}\mathrm{sgn}(c)\bigotimes _{y\in Y(\lambda )}B(\tau 'c(y)), \end{aligned}$$

where we order the Young shape $Y(\lambda )$ by concatenating its rows. Then the matrix set

8 $$\begin{aligned} \{[ u_{\tau ,B}\mid \tau \in T_{\lambda ,m}] ~~\mid ~~ \lambda \vdash n \} \end{aligned}$$

is representative for the natural action of $S_n$ on $V^{\otimes n}$.

## Reduction of the optimization problem

In this section we describe reducing the optimization problem (2) conceptually. In Sect. 4 we consider the reduction computationally. For the remainder of this paper we fix *n* and *q*.

We consider the natural action of $H=S_q^n\rtimes S_n$ on ${\mathbb {R}}^{\mathcal{C}_2}$. If $U_1,\ldots ,U_k$ form a representative set of matrices for this action, then with (4) we obtain a reduction of the size of the optimization problem to polynomial size. To make this reduction explicit in order to apply semidefinite programming, we need to express each $m_i\times m_i$ matrix $U_i^\mathsf{T}M(y)U_i$ as an explicit matrix in which each entry is a linear combination of the variables $y(\omega )$ for $\omega \in \Omega$ (the set of *H*-orbits of $\mathcal{C}_4$).

For $\omega \in \Omega$, let $N_{\omega }$ be the $\mathcal{C}_2\times \mathcal{C}_2$ matrix with 0, 1 entries satisfying

9 $$\begin{aligned} (N_{\omega })_{\{\alpha ,\beta \},\{\gamma ,\delta \}}=1\, \hbox {if and only if}\, \{\alpha ,\beta ,\gamma ,\delta \}\in \omega \end{aligned}$$

for $$\alpha ,\beta ,\gamma ,\delta \in [q]^n$$. Then

10 $$\begin{aligned} U_i^\mathsf{T}M(y)U_i=\sum _{\omega }y(\omega )U_i^\mathsf{T}N_{\omega }U_i. \end{aligned}$$

So to get the reduction, we need to obtain the matrices $U_i^\mathsf{T}N_{\omega }U_i$ explicitly, for each $\omega \in \Omega$ and for each $i=1,\ldots ,k$. We do this in a number of steps.

We first describe in Sect. 3.1 a representative set for the natural action of $S_q$ on ${\mathbb {R}}^{q\times q}$. From this we derive, in Sect. 3.2, with the help of the representative set for the action of $S_n$ on $V^{\otimes n}$ described in Sect. 2.1, a representative set for the action of the wreath product $H=S_q^n\rtimes S_n$ on the set $([q]^n)^2$ of *ordered* pairs of words in $[q]^n$, in other words, on ${\mathbb {R}}^{([q]^n)^2}\cong ({\mathbb {R}}^{q\times q})^{\otimes n}$. From this we derive in Sect. 3.3 a representative set for the action of *H* on the set $\mathcal{C}_2\setminus \{\emptyset \}$ of *unordered* pairs $\{v,w\}$ (including singleton) of words *v*, *w* in $[q]^n$. Then in Sect. 3.4 we derive a representative set for the action of *H* on the set $\mathcal{C}_2^d\setminus \{\emptyset \}$, where $\mathcal{C}_2^d$ is the set of codes in $\mathcal{C}_2$ of minimum distance at least *d*. (So each singleton word belongs to $\mathcal{C}_2^d$.) Finally, in Sect. 3.4 we include the empty set $\emptyset$, by an easy representation-theoretic argument.

### A representative set for the action of $S_q$ on ${\mathbb {R}}^{q\times q}$

We now consider the natural action of $S_q$ on ${\mathbb {R}}^{q\times q}$. Let $e_j$ be the *j*-th unit basis vector in ${\mathbb {R}}^q$, $I_q$ be the $q\times q$ identity matrix, $J_q$ be the all-one $q\times q$ matrix, $\varvec{1}$ be the all-one column vector in ${\mathbb {R}}^q$, $N:=(e_1-e_2)\varvec{1}^\mathsf{T}$, and $E_{i,j}:=e_ie_j^\mathsf{T}$. We furthermore define the following matrices, where we consider matrices in ${\mathbb {R}}^{q\times q}$ as *columns* of the matrices $B_i$:

11 $$\begin{aligned} B_1:= & {} [I_q,J_q-I_q],\nonumber \\ B_2:= & {} [E_{1,1}-E_{2,2}, N-N^\mathsf{T}, N+N^\mathsf{T}-2(E_{1,1}-E_{2,2})] ,\nonumber \\ B_3:= & {} [E_{1,2}+E_{2,3}+E_{3,1}-E_{2,1}-E_{3,2}-E_{1,3}],\nonumber \\ B_4:= & {} [E_{1,3}-E_{3,2}+E_{2,4}-E_{4,1}+E_{3,1}-E_{2,3}+E_{4,2}-E_{1,4}]. \end{aligned}$$

The matrices in ${\mathbb {R}}^{q\times q}$ will in fact be taken as elements of the dual space $({\mathbb {R}}^{q\times q})^*$ (by taking the inner product), so that they are elements of the algebra $\mathcal{O}({\mathbb {R}}^{q\times q})$ of polynomials on the linear space ${\mathbb {R}}^{q\times q}$.

Then $\{B_1,\ldots ,B_4\}$ is representative for the natural action of $S_q$ on ${\mathbb {R}}^{q\times q}$, if $q\ge 4$. If $q\le 3$, we delete $B_4$, and if $q=2$ we moreover delete $B_3$ and the last column of $B_2$ (as this column is 0 if $q=2$). We give a proof in Appendix 1.

If $q\ge 4$, set $k=4$, $m_1=2$, $m_2:=3$, $m_3:=1$, and $m_4:=1$. If $q=3$, set $k=3$, $m_1=2$, $m_2:=3$, and $m_3:=1$. If $q=2$, set $k=2$, $m_1=2$, and $m_2:=2$. For the remainder of this paper we fix *k*, $m_1,\ldots ,m_k$, and $B_1,\ldots ,B_k$.

### A representative set for the action of *H* on $({\mathbb {R}}^{q\times q})^{\otimes n}$

Recall that $H=S_q^n\rtimes S_n$ and that we have fixed *k*, $m_1,\ldots ,m_k$, and $B_1,\ldots ,B_k$ in Sect. 3.1.

We next consider the action of *H* on the set $([q]^n)^2$ of *ordered* pairs of code words. For that, we derive a representative set for the natural action of *H* on $({\mathbb {R}}^{q\times q})^{\otimes n}\cong {\mathbb {R}}^{([q]^n)^2}$ from the results described in Sects. 2.1 and 3.1.

Let ${\varvec{N}}$ be the collection of all *k*-tuples $(n_1,\ldots ,n_k)$ of nonnegative integers adding up to *n*. For $\varvec{n}=(n_1,\ldots ,n_k)\in {\varvec{N}}$, let $\varvec{\lambda \vdash n}$ mean that $\varvec{\lambda }=(\lambda _1,\ldots ,\lambda _k)$ with $\lambda _i\vdash n_i$ for $i=1,\ldots ,k$. (So each $\lambda _i$ is equal to $(\lambda _{i,1},\ldots ,\lambda _{i,t})$ for some *t*.)

For $$\varvec{\lambda }\vdash \varvec{n}$$ define

12 $$\begin{aligned} W_{\varvec{\lambda }}:=T_{\lambda _1,m_1}\times \cdots \times T_{\lambda _k,m_k}, \end{aligned}$$

and for $\varvec{\tau }=(\tau _1,\ldots ,\tau _k)\in W_{\varvec{\lambda }}$ define

13 $$\begin{aligned} v_{\varvec{\tau }}:=\bigotimes _{i=1}^ku_{\tau _i,B_i}. \end{aligned}$$

#### **Proposition 2**

The matrix set

14 $$\begin{aligned} \{[v_{\varvec{\tau }} \mid \varvec{\tau }\in W_{\varvec{\lambda }}] ~~\mid \varvec{n}\in \varvec{N},\varvec{\lambda \vdash n}\} \end{aligned}$$

is representative for the action of $S_q^n\rtimes S_n$ on $({\mathbb {R}}^{q\times q})^{\otimes n}$.

#### *Proof*

Let $L_i$ denote the linear space spanned by $B_i(1),\ldots ,B_i(m_i)$. Then15Now for each ${\varvec{n,\lambda }}$ and $\varvec{\tau },\varvec{\sigma }\in W_{\varvec{\lambda }}$, there is an *H*-isomorphism ${\mathbb {R}}H\cdot v_{\varvec{\tau }}\rightarrow {\mathbb {R}}H\cdot v_{\varvec{\sigma }}$ bringing $v_{\varvec{\tau }}$ to $v_{\varvec{\sigma }}$, since for each $i=1,\ldots ,k$, setting $H_i:=S_q^{n_i}\rtimes S_{n_i}$, there is an $H_i$-isomorphism ${\mathbb {R}}H_i\cdot u_{\tau _i,B_i}\rightarrow {\mathbb {R}}H_i\cdot u_{\sigma _i,B_i}$. Hence (where ${{\mathrm{Sym}}_t(X):=(X^{\otimes t})^{S_t}}$ for any $t\in {\mathbb {Z}}_+$ and linear space *X*, with the natural action of $S_t$ on $X^{\otimes t}$)16as $\sum _{i=1}^km_i^2=\dim ({\mathbb {R}}^{q\times q}\otimes {\mathbb {R}}^{q\times q})^{S_q}$. So we have equality throughout in (16), and hence each ${\mathbb {R}}H\cdot v_{\varvec{\tau }}$ is irreducible, and if $\varvec{\lambda }\ne \varvec{\lambda '}$, then for each ${\varvec{\tau }}\in W_{\varvec{\lambda }}$ and ${\varvec{\tau '}}\in W_{\varvec{\lambda '}}$, ${\mathbb {R}}H\cdot v_{\varvec{\tau }}$ and ${\mathbb {R}}H\cdot v_{\varvec{\tau '}}$ are not *H*-isomorphic. $\square$

### Unordered pairs

We now go over from the set $([q]^n)^2$ of ordered pairs of code words to the set $\mathcal{C}_2\setminus \{\emptyset \}$ of unordered pairs (including singletons) of code words. For this we consider the action of the group $S_2$ on ${\mathbb {R}}^{[q]^n\times [q]^n}\cong {\mathbb {R}}^{([q]^n)^2}\cong ({\mathbb {R}}^{q\times q})^{\otimes n}$, where the nonidentity element $\sigma$ in $S_2$ acts as taking the transpose. The actions of $S_2$ and *H* commute.

Let *F* be the $(\mathcal{C}_2\setminus \{\emptyset \})\times ([q]^n)^2$ matrix with 0, 1 entries satisfying

17 $$\begin{aligned} F_{\{\alpha ,\beta \},(\gamma ,\delta )}=1 \hbox {if and only if} \{\gamma ,\delta \}=\{\alpha ,\beta \}, \end{aligned}$$

for $\alpha ,\beta ,\gamma ,\delta \in [q]^n$. Then the function $x\mapsto Fx$ is an *H*-isomorphism $({\mathbb {R}}^{([q]^n)^2})^{S_2}\rightarrow {\mathbb {R}}^{\mathcal{C}_2\setminus \{\emptyset \}}$.

Now note that each $B_i(j)$, as matrix in ${\mathbb {R}}^{q\times q}$, is $S_2$-invariant (i.e., symmetric) except for $B_2(2)$ and $B_3(1)$, while $\sigma \cdot B_2(2)=-B_2(2)$ and $\sigma \cdot B_3(1)=-B_3(1)$ (as $B_2(2)$ and $B_3(1)$ are skew-symmetric). So for any ${\varvec{n}}\in \varvec{N}$, $\varvec{\lambda \vdash n}$, and $\varvec{\tau }\in W_{\varvec{\lambda }}$, we have

18 $$\begin{aligned} \sigma \cdot v_{\varvec{\tau }} = (-1)^{|\tau _2^{-1}(2)|+|\tau _3^{-1}(1)|} v_{\varvec{\tau }}. \end{aligned}$$

Therefore, let $W'_{\varvec{\lambda }}$ be the set of those $\varvec{\tau }\in W_{\varvec{\lambda }}$ with $|\tau _2^{-1}(2)|+|\tau _3^{-1}(1)|$ even. Then the matrix set

19 $$\begin{aligned} \{[ v_{\varvec{\tau }} \mid \varvec{\tau }\in W'_{\varvec{\lambda }} ] ~~\mid ~~ \varvec{n}\in \varvec{N},\varvec{\lambda \vdash n}\} \end{aligned}$$

is representative for the action of *H* on $({\mathbb {R}}^{([q]^n)^2})^{S_2}$. Hence the matrix set

20 $$\begin{aligned} \{[Fv_{\varvec{\tau }} \mid \varvec{\tau }\in W'_{\varvec{\lambda }} ] ~~{\mid }~~ \varvec{n}\in \varvec{N}, \varvec{\lambda \vdash n}\} \end{aligned}$$

is representative for the action of *H* on ${\mathbb {R}}^{\mathcal{C}_2\setminus \{\emptyset \}}$.

### Restriction to pairs of words at distance at least *d*

Let $d\in {\mathbb {Z}}_+$, and let $\mathcal{C}^d_2$ be the collection of elements of $\mathcal{C}_2$ of minimum distance at least *d*. Note that each singleton code word belongs to $\mathcal{C}_2^d$, and that *H* acts on $\mathcal{C}^d_2$. From (20) we derive a representative set for the action of *H* on ${\mathbb {R}}^{\mathcal{C}_2^d\setminus \{\emptyset \}}$.

To see this, let for each $t\in {\mathbb {Z}}_+$, $L_t$ be the subspace of ${\mathbb {R}}^{\mathcal{C}_2}$ spanned by the elements $e_{\{\alpha ,\beta \}}$ with $\alpha ,\beta \in [q]^n$ and $d_H(\alpha ,\beta )=t$. (For any $Z\in \mathcal{C}_2$, $e_Z$ denotes the unit base vector in ${\mathbb {R}}^{\mathcal{C}_2^d}$ for coordinate *Z*.)

Then for any ${\varvec{n}\in \varvec{N}}$, ${\varvec{\lambda \vdash n}}$, and $\varvec{\tau }\in W'_{\varvec{\lambda }}$, the irreducible representation $H\cdot Fv_{\varvec{\tau }}$ is contained in $L_t$, where

21 $$\begin{aligned} t:=n-|\tau _1^{-1}(1)|-|\tau _2^{-1}(1)|, \end{aligned}$$

since $B_1(1)=I_q$ and $B_2(1)=E_{1,1}-E_{2,2}$ are the only two entries $B_i(j)$ in the $B_i$ that have nonzeros on the diagonal of the matrix $B_i(j)$. Let $W''_{\varvec{\lambda }}$ be the set of those $\varvec{\tau }$ in $W'_{\varvec{\lambda }}$ with

22 $$\begin{aligned} n-|\tau _1^{-1}(1)|-|\tau _2^{-1}(1)|\in \{0,d,d+1,\ldots ,n\}. \end{aligned}$$

Then a representative set for the action of *H* on $\mathcal{C}^d_2\setminus \{\emptyset \}$ is

23 $$\begin{aligned} \big \{[Fv_{\varvec{\tau }} \mid \varvec{\tau }\in W''_{\varvec{\lambda }} ] ~~\mid ~~ \varvec{n}\in \varvec{N}, \varvec{\lambda \vdash n} \big \}. \end{aligned}$$

### Adding $\emptyset$

To obtain a representative set for the action of *H* on $\mathcal{C}^d_2$, note that *H* acts trivially on $\emptyset$. So $e_{\emptyset }$ belongs to the *H*-isotypical component of ${\mathbb {R}}^{\mathcal{C}_2}$ that consists of *H*-invariant elements. Now the *H*-isotypical component of ${\mathbb {R}}^{\mathcal{C}_2\setminus \{\emptyset \}}$ that consists of the *H*-invariant elements corresponds to the matrix in the representative set indexed by indexed by $\varvec{n}=(n,0,0,0)$ and $\varvec{\lambda }=((n),(),(),())$, where $()\vdash 0$. So to obtain a representative set for ${\mathbb {R}}^{\mathcal{C}_2}$, we just add $e_{\emptyset }$ as column to this matrix.

## How to compute $(Fv_{\varvec{\tau }})^\mathsf{T}N_{\omega }Fv_{\varvec{\sigma }}$

We now have a reduction of the original problem to blocks with coefficients $(Fv_{\varvec{\tau }})^\mathsf{T}N_{\omega }Fv_{\varvec{\sigma }}$, for $\varvec{n}\in \varvec{N}$, $\varvec{\lambda \vdash n}$, $\varvec{\tau },\varvec{\sigma }\in W_{\varvec{\lambda }}$, and $\omega \in \Omega$. The number and orders of these blocks are bounded by a polynomial in *n*, but computing these coefficients still must be reduced in time, since the order of *F*, $v_{\varvec{\tau }}$, $v_{\varvec{\sigma }}$, and $N_{\omega }$ is exponential in *n*.

Fix $\varvec{n}\in \varvec{N}$, $\varvec{\lambda \vdash n}$, and $\varvec{\tau },\varvec{\sigma }\in W_{\varvec{\lambda }}$. For any $\omega \in \Omega$, let $L_{\omega }:=F^\mathsf{T}N_{\omega }F$. So $L_{\omega }$ is a $([q]^n\times [q]^n)\times ([q]^n\times [q]^n)$ matrix with 0,1 entries satisfying

24 $$\begin{aligned} (L_{\omega })_{(\alpha ,\beta ),(\gamma ,\delta )}=1 ~ \hbox {if and only if}~ \{\alpha ,\beta ,\gamma ,\delta \}\in \omega , \end{aligned}$$

for all $\alpha ,\beta ,\gamma ,\delta \in [q]^n$. By definition of $L_{\omega }$,

25 $$\begin{aligned} (Fv_{\varvec{\tau }})^\mathsf{T}N_{\omega }Fv_{\varvec{\sigma }} = v_{\varvec{\tau }}^\mathsf{T}L_{\omega }v_{\varvec{\sigma }}. \end{aligned}$$

So it suffices to evaluate the latter value.

Let $\Pi$ be the collection of partitions of $\{1,2,3,4\}$ into at most *q* parts. There is the following bijection between $\Pi$ and the set of orbits of the action of $S_q$ on $[q]^4$.

For each word $w\in [q]^4$, let ${\mathrm{part}(w)}$ be the partition $P\in \Pi$ such that *i* and *j* belong to the same class of *P* if and only if $w_i=w_j$ (for $i,j=1,\ldots ,4$). Then two elements $v,w\in [q]^4$ belong to the same $S_q$-orbit if and only if ${\mathrm{part}(v)=\mathrm{part}(w)}$. Note that $|\Pi |=8$ if $q=2$, $|\Pi |=14$ if $q=3$, and $|\Pi |=15$ if $q\ge 4$. (In all cases, $|\Pi |=\dim ({\mathbb {R}}^{q\times q})^{S_q}=\sum _{i=1}^km_i^2$.)

For $$P\in \Pi $$, let

26 $$\begin{aligned} d_P:={\mathop {\mathop {\sum }\limits _{i_1,\ldots ,i_4\in [q]}}\limits _{\mathrm{part}{i_1\cdots i_4}=P}}e_{i_1}e_{i_2}^\mathsf{T}\otimes e_{i_3}e_{i_4}^\mathsf{T}, \end{aligned}$$

where each $e_i$ is a unit basis column vector in ${\mathbb {R}}^q$, so that $e_ie_j^\mathsf{T}$ is a matrix in ${\mathbb {R}}^{q\times q}$. Then $D:=\{d_P\mid P\in \Pi \}$ is a basis of $({\mathbb {R}}^{q\times q}\otimes {\mathbb {R}}^{q\times q})^{S_q}$. Let $D^*$ be the dual basis.

For any $(\alpha ,\beta ,\gamma ,\delta )\in ([q]^n)^4$, let

27 $$\begin{aligned} \psi (\alpha ,\beta ,\gamma ,\delta ):=\prod _{i=1}^nd^*_{\mathrm{part}(\alpha _i\beta _i\gamma _i\delta _i)}, \end{aligned}$$

which is a degree *n* polynomial on $({\mathbb {R}}^{q\times q}\otimes {\mathbb {R}}^{q\times q})^{S_q}$. Then $\psi (\alpha ,\beta ,\gamma ,\delta )=\psi (\alpha ',\beta ',\gamma ',\delta ')$ if and only if $(\alpha ,\beta ,\gamma ,\delta )$ and $(\alpha ',\beta ',\gamma ',\delta ')$ belong to the same *H*-orbit on $([q]^n)^4$. So this gives a bijection between the set *Q* of degree *n* monomials expressed in the dual basis $D^*$ and the set of *H*-orbits on $([q]^n)^4\cong ([q]^4)^n$. The function $([q]^n)^4\rightarrow \mathcal{C}_4$ with $(\alpha _1,\ldots ,\alpha _4)\mapsto \{\alpha _1,\ldots ,\alpha _4\}$ then gives a surjective function $\omega :Q\rightarrow \Omega \setminus \{\{\emptyset \}\}$.

For any $\mu \in Q$, define

28 $$\begin{aligned} K_{\mu }:={\mathop {\mathop {\sum }_{d_1,\ldots ,d_n\in D}}\limits _{d_{1}^*\cdots d_{n}^*=\mu }}\bigotimes _{j=1}^{n}d_j. \end{aligned}$$

### **Lemma 1**

For each $$\omega \in \Omega $$: $$\displaystyle L_{\omega }={\mathop {\mathop {\sum }\nolimits _{\mu \in Q}}\limits _{\omega (\mu )=\omega }}K_{\mu }$$.

### *Proof*

Choose $$\alpha ,\beta ,\gamma ,\delta \in [q]^n$$. Then

29 $$\begin{aligned} {\mathop {\mathop {\sum }_{\mu \in Q}}\limits _{\omega (\mu )=\omega }} (K_{\mu })_{(\alpha ,\beta ),(\gamma ,\delta )}= & {} {\mathop {\mathop {\sum }_{\mu \in Q}}\limits _{\omega (\mu )=\omega }}\quad {\mathop {\mathop {\sum }_{P_1,\ldots ,P_n\in \Pi }}\limits _{d^*_{P_1}\cdots d^*_{P_n}=\mu }} \left( \bigotimes _{i=1}^nd_{P_i}\right) _{\alpha ,\beta ,\gamma ,\delta } \nonumber \\= & {} {\mathop {\mathop {\sum }_{\mu \in Q}}\limits _{\omega (\mu )=\omega }}\quad {\mathop {\mathop {\sum }_{P_1,\ldots ,P_n\in \Pi }}\limits _{d^*_{P_1}\cdots d^*_{P_n}=\mu }} \prod _{i=1}^n(d_{P_i})_{\alpha _i,\beta _i,\gamma _i,\delta _i}. \end{aligned}$$

Now the latter value is 1 if $\omega (d^*_{\mathrm{part}(\alpha _1\beta _1\gamma _1\delta _1)}\cdots d^*_{\mathrm{part}(\alpha _n\beta _n\delta _n\gamma _n)})=\omega$, and is 0 otherwise. So it is equal to $(L_{\omega })_{(\alpha ,\beta ),(\gamma ,\delta )}$. $\square$

By this lemma, it suffices to compute $v_{\varvec{\tau }}^\mathsf{T}K_{\mu }v_{\varvec{\sigma }}$ for each $\mu \in Q$. To this end, define the following degree *n* polynomial on $W:=({\mathbb {R}}^{q\times q}\otimes {\mathbb {R}}^{q\times q})^{S_q}$:

30 $$\begin{aligned} p_{\varvec{\tau ,\sigma }}:= \prod _{i=1}^k {\mathop {\mathop {\sum }_{\tau _i'\sim \tau _i}}\limits _{\sigma _i'\sim \sigma _i}}\sum _{c_i,c_i'\in C_{\lambda _i}}\mathrm{sgn}(c_ic_i') \prod _{y\in Y(\lambda _i)} B_i(\tau _i'c_i(y))\otimes B_i(\sigma _i'c_i'(y)). \end{aligned}$$

This polynomial can be computed (i.e., expressed as linear combination of monomials in $B_i(j)\otimes B_i(h)$) in time bounded by a polynomial in *n* (Gijswijt [5], see Appendix 2 in Sect. 1 below).

### **Lemma 2**

$$\displaystyle \sum _{\mu \in Q}(v_{\varvec{\tau }}^\mathsf{T}K_{\mu }v_{\varvec{\sigma }})\mu = p_{\varvec{\tau ,\sigma }}$$.

### *Proof*

We can write for each $\mu \in Q$:

31 $$\begin{aligned} v_{\varvec{\tau }}^\mathsf{T}K_{\mu }v_{\varvec{\sigma }} = (v_{\varvec{\tau }}\otimes v_{\varvec{\sigma }})(K_{\mu }), \end{aligned}$$

using the fact that $v_{\varvec{\tau }},v_{\varvec{\sigma }}\in (({\mathbb {R}}^{q\times q})^{\otimes n})^*$ and $K_{\mu }\in ({\mathbb {R}}^{q\times q})^{\otimes n}\otimes ({\mathbb {R}}^{q\times q})^{\otimes n}$. So it suffices to show

32 $$\begin{aligned} \sum _{\mu \in Q}(v_{\varvec{\tau }}\otimes v_{\varvec{\sigma }})(K_{\mu })\mu =p_{\varvec{\tau ,\sigma }}. \end{aligned}$$

Consider any $f=f_1\cdots f_n$ with $f_j\in W^*$ for $j=1,\ldots ,n$. Then

33 $$\begin{aligned} f=\sum _{\mu \in Q}\left( \bigotimes _{j=1}^{n}f_j)(K_{\mu }\right) \mu . \end{aligned}$$

Indeed,

34 $$\begin{aligned} \sum _{\mu \in Q}\left( \bigotimes _{j=1}^nf_j)(K_{\mu }\right) \mu= & {} {\mathop {\mathop {\sum }_{d_1,\ldots ,d_n\in D}}\limits _{d_1^*\cdots d_n^*=\mu }}\left( \bigotimes _{j=1}^nf_j\right) \left( \bigotimes _{j=1}^n d_j\right) \mu \nonumber \\= & {} \sum _{d_1,\ldots ,d_n\in D}\prod _{j=1}^nf_j(d_j)d_j^* =\prod _{j=1}^n\sum _{d\in D}f_j(d)d^*\nonumber \\= & {} \prod _{j=1}^nf_j=f. \end{aligned}$$

Applying (33) to each term *f* of $p_{\varvec{\tau ,\sigma }}$ as given by (30) we obtain (32), in view of (7) and (13). $\square$

So $v_{\varvec{\tau }}^\mathsf{T}K_{\mu }v_{\varvec{\sigma }}$ can be computed by expressing the polynomial $p_{\varvec{\tau ,\sigma }}$ as linear combination of monomials $\mu \in Q$, which are products of linear functions in $D^*$. So it suffices to express each $B_i(j)\otimes B_i(h)$ as linear function into the basis $D^*$, that is, to calculate the numbers $(B_i(j)\otimes B_i(h))(d_P)$ for all $i=1,\ldots ,k$, $j,h=1,\ldots ,m_i$, and $P\in \Pi$—see Appendix 3 (Sect. 1 below).

We finally consider the entries in the row and column for $\emptyset$ in the matrix associated with $\varvec{\lambda }=((n),(),(),())$ (cf. Sect. 3.5). Trivially, $e_{\emptyset }^\mathsf{T}M(x)e_{\emptyset }=(M(x))_{\emptyset ,\emptyset } =x(\emptyset )$, which is set to 1 in the optimization problem. Any $\varvec{\tau }\in W_{\varvec{\lambda }}$ is determined by the number *t* of 2’s in the row of the Young shape *Y*((*n*)). Then

35 $$\begin{aligned} v_{\varvec{\tau }}={\mathop {\mathop {\sum }_{u,w\in [q]^n}}\limits _{d_H(u,w)=t}}e_{(u,w)} \text {~~~and ~~hence~~~} Fv_{\varvec{\tau }}={\mathop {\mathop {\sum }_{u,w\in [q]^n}}\limits _{d_H(u,w)=t}}e_{\{u,w\}}. \end{aligned}$$

Hence, as $$\emptyset \cup \{u,w\}=\{u,w\}$$,

36 $$\begin{aligned} e_{\emptyset }^\mathsf{T}M(x)Fv_{\varvec{\tau }} ={\mathop {\mathop {\sum }_{u,w\in [q]^n}}\limits _{d_H(u,w)=t}}x({\{u,w\}}) ={{n}\atopwithdelims (){t}}q^n(q-1)^ty(\omega ), \end{aligned}$$

where $\omega$ is the *H*-orbit of $\mathcal{C}_4$ consisting of all pairs $\{\alpha ,\beta \}$ with $d_H(\alpha ,\beta )=t$.

## Appendix 1: The representative set $\{B_1,\ldots ,B_k\}$ for the action of $S_q$ on ${\mathbb {C}}^{q\times q}$

In this section we show that the matrix set $\{B_1,\ldots ,B_k\}$ as given in (11) is representative for the natural action of $S_q$ on ${\mathbb {C}}^{q\times q}$. For $a\in {\mathbb {C}}^q$, let $\Delta _a$ be the $q\times q$ diagonal matrix with diagonal *a*, and let $\varvec{1}$ be the all-one column vector in ${\mathbb {C}}^q$. Define

37 $$\begin{aligned} V_{1,1}:= & {} \{\lambda I_q\mid \lambda \in {\mathbb {C}}\},\nonumber \\ V_{1,2}:= & {} \{\lambda (J_q-I_q)\mid \lambda \in {\mathbb {C}}\},\nonumber \\ V_{2,1}:= & {} \{\Delta _a\mid a\in {\mathbb {C}}^q, a^\mathsf{T}\varvec{1}=0\},\nonumber \\ V_{2,2}:= & {} \{a\varvec{1}^\mathsf{T}-\varvec{1}a^\mathsf{T}\mid a\in {\mathbb {C}}^q, a^\mathsf{T}\varvec{1}=0\},\nonumber \\ V_{2,3}:= & {} \{a\varvec{1}^\mathsf{T}+\varvec{1}a^\mathsf{T}-2\Delta _a\mid a\in {\mathbb {C}}^q, a^\mathsf{T}\varvec{1}=0\},\nonumber \\ V_{3,1}:= & {} \{X\in {\mathbb {C}}^{q\times q}\mid X\text { skew-symmetric}, X\varvec{1}=0\},\nonumber \\ V_{4,1}:= & {} \{X\in {\mathbb {C}}^{q\times q}\mid X\text { symmetric}, X\varvec{1}=0, X_{i,i}=0\text { for all }i\in [q]\}. \end{aligned}$$

Observe that each $V_{i,j}$ is $S_q$-stable, and that $V_{i,j}$ and $V_{i',j'}$ are orthogonal whenever $(i,j)\ne (i',j')$ (with respect to the inner product $X,Y\mapsto \text {tr}(X^*Y)$). Moreover $\lambda I_q\mapsto \lambda (J_q-I_q)$ gives an $S_q$-isomorphism $V_{1,1}\rightarrow V_{1,2}$, $\Delta _a\mapsto a\varvec{1}^\mathsf{T}-\varvec{1}a^\mathsf{T}$ gives an $S_q$-isomorphism $V_{2,1}\rightarrow V_{2,2}$, and $\Delta _a\mapsto a\varvec{1}^\mathsf{T}+\varvec{1}a^\mathsf{T}-2\Delta _a$ gives an $S_q$-isomorphism $V_{2,1}\rightarrow V_{2,3}$.

Let $q\ge 4$. Then $\dim (V_{i,j})>0$ for all *i*, *j*. Set, as before, $m_1=2$, $m_2=3$, $m_3=m_4=1$. Then $\sum _{i=1}^4m_i^2=15$, which is equal to the number of partitions of $\{1,2,3,4\}$, hence to the dimension of $({\mathbb {C}}^{q\times q}\otimes {\mathbb {C}}^{q\times q})^{S_q}$. This implies that the $V_{i,j}$ in fact form an orthogonal *decomposition* of ${\mathbb {C}}^{q\times q}$ into *irreducible* representations and that $V_{i,j}$ and $V_{i',j'}$ are equivalent representations if and *only if* $i=i'$ (as any further representation, or decomposition, or equivalence would yield that the sum of the squares of the multiplicities of the irreducible representations is strictly larger than 15, contradicting the fact that $\Phi$ in (4) is bijective).

Now $B_{1,1}$ and $B_{1,2}$ are the elements of $V_{1,1}$ and $V_{1,2}$ with $\lambda =1$. Moreover, $B_{2,1}$, $B_{2,2}$, and $B_{2,3}$ are the elements of $V_{2,1}$, $V_{2,2}$, and $V_{2,3}$ with $a=e_1-e_2$. Finally, $B_{3,1}$ and $B_{4,1}$ are nonzero elements of $V_{3,1}$ and $V_{4,1}$ (they can be chosen arbitrarily). This implies that $\{B_1,\ldots ,B_4\}$ is a representative matrix set.

If $q=3$, then $\dim (V_{4,1})=0$, while the dimension of $({\mathbb {C}}^{3\times 3}\otimes {\mathbb {C}}^{3\times 3})^{S_3}$ is equal to the number of partitions of $\{1,2,3,4\}$ into at most 3 classes, which is $2^2+3^2+1^2=14$. If $q=2$, then moreover $\dim (V_{2,3})=\dim (V_{3,1})=0$, while the dimension of $({\mathbb {C}}^{2\times 2}\otimes {\mathbb {C}}^{2\times 2})^{S_2}$ is equal to the number of partitions of $\{1,2,3,4\}$ into at most 2 classes, which is $2^2+2^2=8$. Similarly as above, this implies that also for $q\le 3$, $B_1,\ldots ,B_k$ form a representative matrix set.

## Appendix 2: Computation of $p_{\tau ,\sigma }$

For any $n,m\in {\mathbb {Z}}_+$, $\lambda \vdash n$, and $\tau ,\sigma \in T_{\lambda ,m}$, define the polynomial $p_{\tau ,\sigma }\in {\mathbb {R}}[x_{j,h}\mid j,h=1,\ldots ,m]$ by

38 $$\begin{aligned} p_{\tau ,\sigma }(X):= {\mathop {\mathop {\sum }_{\tau '\sim \tau }}\limits _{\sigma '\sim \sigma }}\sum _{c,c'\in C_{\lambda }}\mathrm{sgn}(cc') \prod _{y\in Y(\lambda )} x_{\tau 'c(y),\sigma 'c'(y)}, \end{aligned}$$

for $$X=(x_{j,h})_{j,h=1}^m\in {\mathbb {R}}^{m\times m}$$.

### **Proposition 3**

Expressing $p_{\tau ,\sigma }$ as a linear combination of monomials can be done in polynomial time, for fixed *m*.

### *Proof*

First observe that

39 $$\begin{aligned} p_{\tau ,\sigma }(X)= & {} |C_{\lambda }|{\mathop {\mathop {\sum }_{\tau '\sim \tau }}\limits _{\sigma '\sim \sigma }}\sum _{c\in C_{\lambda }}\mathrm{sgn}(c) \prod _{y\in Y_{\lambda }}x_{\tau '(y),\sigma 'c(y)}\nonumber \\= & {} |C_{\lambda }| {\mathop {\mathop {\sum }_{\tau '\sim \tau }}\limits _{\sigma '\sim \sigma }}\prod _{j=1}^{\lambda _1}\det ((x_{\tau '(i,j),\sigma '(i',j)})_{i,i'=1}^{\lambda ^*_j}). \end{aligned}$$

($\lambda ^*$ is the dual partition of $\lambda$; that is, $\lambda ^*_j$ is the height of column *j*.)

For fixed *m*, when *n* grows, there will be several columns of $Y(\lambda )$ that are the same both in $\tau '$ and in $\sigma '$. More precisely, for given $\tau ',\sigma '$ let the ‘count function’ $\kappa$ be defined as follows: for $t\in {\mathbb {Z}}_+$ and $v,w\in [m]^t$, $\kappa (v,w)$ is the number of columns *j* of height *t* such that $\tau '(i,j)=v_i$ and $\sigma '(i,j)=w_i$ for all $i=1,\ldots ,t$. Then for each $i\le h:=\mathrm{height}(\lambda )$ and each $s\in [m]$:

40 $$\begin{aligned} \displaystyle \sum _{t=i}^{h}{\mathop {\mathop {\sum }_{v,w\in [m]^t}}\limits _{v_i=s}}\kappa (v,w)= & {} \hbox {number of } s \hbox { in row } i \hbox { of } \tau , \hbox {and}\nonumber \\ \displaystyle \sum _{t=i}^{h}{\mathop {\mathop {\sum }_{v,w\in [m]^t}}\limits _{w_i=s}}\kappa (v,w)= & {} \hbox {number of } s \hbox { in row } i \hbox { of } \sigma . \end{aligned}$$

For any given function $\kappa :\bigcup _{i=1}^{h}[m]^i\times [m]^i\rightarrow {\mathbb {Z}}_+$ satisfying (40), there are precisely

41 $$\begin{aligned} \prod _{t=1}^h\frac{(\lambda _t-\lambda _{t+1})!}{\prod _{v,w\in [m]^t}\kappa (v,w)!} \end{aligned}$$

pairs $\tau '\sim \tau$ and $\sigma '\sim \sigma$ having count function $\kappa$ (setting $\lambda _{h+1}:=0$). (Note that (40) implies $\lambda _t-\lambda _{t+1}=\sum _{v,w\in [m]^t}\kappa (v,w)$, for each *t*, so that for each *t*, the factor in (41) is a Newton multinomial coefficient.) Hence

42 $$\begin{aligned} p_{\tau ,\sigma } =|C_{\lambda }| \sum _{\kappa }\prod _{t=1}^h(\lambda _t-\lambda _{t+1})!\prod _{v,w\in [m]^t} \frac{\det ((x_{v(i),w(i')})_{i,i'=1}^t)^{\kappa (v,w)}}{\kappa (v,w)!}, \end{aligned}$$

where $\kappa$ ranges over functions $\kappa :\bigcup _{t=1}^h([m]^t\times [m]^t)\rightarrow {\mathbb {Z}}_+$ satisfying (40). $\square$

## Appendix 3: Expressing $B_i(j)\otimes B_i(h)$ into $d^*_P$

Recall that each $B_i(j)$ is a linear function on ${\mathbb {R}}^{q\times q}$, and that each $d_P$ is an element of ${\mathbb {R}}^{q\times q}\otimes {\mathbb {R}}^{q\times q}$, where *P* belongs to the set $\Pi$ of partitions of $\{1,\ldots ,4\}$ with at most *q* classes. We express each $B_i(j)\otimes B_i(h)$ in the dual basis $B^*:=\{d^*_P\mid P\in \Pi \}$. The coefficient of $d^*_P$ is obtained by evaluating $(B_i(j)\otimes B_i(h))(d_P)$. This is routine, but we display the expressions.

For this, denote any subset *X* of $\{1,\ldots ,4\}$ by a string formed by the elements of *X*, and denote a partition *P* of $\{1,\ldots ,4\}$ by a sequence of its classes (for instance, $d^*_{13,2,4}$ denotes the dual variable $d^*_P$ associated with partition $P=\{\{1,3\},\{2\},\{4\}\}$ of $\{1,2,3,4\}$). Then:

$$\begin{aligned} B_1(1)\otimes B_1(1)= & {} q d^*_{1234} +q(q-1) d^*_{12,34},\\ B_1(1)\otimes B_1(2)= & {} q(q-1)(d^*_{123,4} + d^*_{124,3} +(q-2) d^*_{12,3,4}),\\ B_1(2)\otimes B_1(1)= & {} q(q-1)(d^*_{1,234} + d^*_{134,2} +(q-2) d^*_{1,2,34}),\\ B_1(2)\otimes B_1(2)= & {} q(q-1)(d^*_{13,24} + d^*_{14,23} +(q-2)(d^*_{13,2,4} + d^*_{14,2,3} + d^*_{1,23,4}\\&+ \,d^*_{1,24,3} +(q-3) d^*_{1,2,3,4})).\\ B_2(1)\otimes B_2(1)= & {} 2 d^{*}_{1234} -2 d^*_{12,34},\\ B_2(1)\otimes B_2(2)= & {} 2q(d^*_{123,4} - d^*_{124,3}),\\ B_2(1)\otimes B_2(3)= & {} 2(q-2)(d^*_{124,3} + d^*_{123,4} -2 d^*_{12,3,4}),\\ B_2(2)\otimes B_2(1)= & {} 2q(d^*_{134,2} - d^*_{1,234}),\\ B_2(2)\otimes B_2(2)= & {} 2q(2d^*_{13,24} -2 d^*_{14,23} +(q-2)(d^*_{13,2,4} - d^*_{14,2,3} - d^*_{1,23,4} + d^*_{1,24,3})),\\ B_2(2)\otimes B_2(3)= & {} 2q(q-2)(d^*_{13,2,4} + d^*_{14,2,3} - d^*_{1,23,4} - d^*_{1,24,3}),\\ B_2(3)\otimes B_2(1)= & {} 2(q-2)(d^*_{1,234} + d^*_{134,2} -2 d^*_{1,2,34}),\\ B_2(3)\otimes B_2(2)= & {} 2q(q-2)(d^*_{13,2,4} - d^*_{14,2,3} + d^*_{1,23,4} - d^*_{1,24,3}),\\ B_2(3)\otimes B_2(3)= & {} 2(q-2)(2 d^*_{13,24} +2 d^*_{14,23} +(q-4)(d^*_{13,2,4} + d^*_{14,2,3} + d^*_{1,23,4}\\&+\, d^*_{1,24,3}) -4(q-3) d^*_{1,2,3,4}).\\ B_3(1)\otimes B_3(1)= & {} 6(d^*_{13,24} - d^*_{14,23} - d^*_{13,2,4} + d^*_{14,2,3} + d^*_{1,23,4} - d^*_{1,24,3}).\\ B_4(1)\otimes B_4(1)= & {} 8(d^*_{13,24} + d^*_{14,23} - d^*_{13,2,4} - d^*_{14,2,3} - d^*_{1,23,4} - d^*_{1,24,3}) +16 d^*_{1,2,3,4}. \end{aligned}$$
